# Correlation between histocompatibility antigens and recurrent aphthous stomatitis in the brazilian population

**DOI:** 10.1016/S1808-8694(15)30662-5

**Published:** 2015-10-19

**Authors:** Niels Salles Willo Wilhelmsen, Raimar Weber, Francisco Monteiro, Jorge Kalil, Ivan Dieb Miziara

**Affiliations:** 1 PhD in Otorhinolaryngology; 2 Graduate Student (PhD) in Otorhinolaryngology; 3 PhD in Nephrology, Professor; 4 Full Professor of Immunology and Clinical Allergy of the University of São Paulo Medical School; 5 Associate Professor of Otorhinolaryngology. University of São Paulo Medical School

**Keywords:** hla antigens, aphthous stomatitis, genetics, stomatology

## Abstract

Recurrent aphthous stomatitis is a common oral mucosa disorder that affects 20% of the world's population, characterized by recurring painful ulcers in the mouth. The diagnosis is primarily based on the patient's clinical history. Inheritance may pose as a risk factor for the disease; however, the studies available are inconclusive as to the results attained, and they vary according to the population studied.

**Aim:**

to typify class I and class II HLA molecules and to assess how frequent these molecules are present in the Brazilian population with Recurrent Aphthous Stomatitis, compared to healthy controls.

**Materials and Methods:**

In this prospective, cross-sectional and investigative study, thirty one patients with diagnostic hypothesis of recurrent aphthous stomatitis were seen from February of 2004 to May of 2006. We obtained the DNA from those patients who matched the inclusion criteria and typified their HLA by PCR.

**Results:**

In those patients with Recurrent Minor Aphthous Stomatitis we found statistically significant occurrences of HLA-A33 and HLA-B35.

**Conclusion:**

HLA-A33 and HLA-B35 may be associated with recurrent minor aphthous stomatitis in the Brazilian's population.

## INTRODUCTION

Recurrent Aphthoid Stomatitis (RAS) is a common disease that causes the appearance of recurrent aphtha in the oral mucosa. Its incidence is of about 20% in the world population. It is more frequent in females. Despite the high incidence, and many studies dedicated to unveiling its causes, it still is very controversial regarding etiology^1^.

RAS is currently defined as a disease characterized by aphtha lesions in the oral mucosa, in a recurrent fashion (every fortnight or monthly), for a minimum period of one year, and its onset is usually in childhood or adolescence^2^, without evidences of associated systemic diseases. Nonetheless, some authors state that the incidence of RAS is greater in the second decade of life^3^,^4^.

The disease usually has three clinical forms, based on the aspect and size of the ulcerations: minor, major and herpetiform. Recurrent Aphthoid Stomatitis Minor (RASi) affects 80% of the patients with RAS, and it is characterized by a shallow painful, oval or round-shaped ulcer, smaller than 5mm in diameter. It affects the non-keratinized oral mucosa, especially vestibule, labial and oral mucosa, soft palate, tongue and tongue floor. The lesions heal within 7 to 10 days. Recurrent Aphthoid Stomatitis Major (RASj) is the most severe form. Aphtha diameter can get bigger than 1cm, remaining in the oral cavity for a period longer than two weeks, often times leaving a scar. The third form of the disease, and the less common one is Herpetiform Recurrent Aphthoid Stomatitis (HRAS), characterized by multiple and painful 1 to 3mm ulcers, which may coalesce, forming larger and irregular lesions^5^.

Family history seems to be relevant in the genesis of RAS, and reports of cases in the same family are found in 24% to 46% of the times^6^,^7^. Moreover, patients with family history of RAS can develop ulcers earlier on and have more severe manifestations than those without family history^8^. Monozygotic twins have a greater likelihood of suffering the disease when compared to dizygotic twins^9^.

Numerous associations and non-associations of HLA and RAS antigen have been reported in the medical literature. The association between the disease and HLA-B12 was described by Lehner et al.^10^ and Malmström et al.11, however it was not confirmed by other authors^12^,^13^. In groups of patients of different ethnical origin, a significant association between HLA-DR2 and RAS was noticed^10^,^14^.

RAS pathophysiology seems to be associated with a disorder in immunomodulation^15^. Lymphocytes seem to be the predominant cells in aphthoid lesions, and there was a variation in the CD4+/CD8+ ratio during its different stages - prodrome or pre-ulceration, ulceration and healing^16^,^17^,^18^.

The main role played by the major histocompatibility complex in humans, called HLA system is to present processed peptides for recognition by the T-cell receptor in the different immunological functions. Since such recognition is made at the same time involving the peptide and the HLA molecule, small variations in one of the two components cause immunologic recognition and the triggering of cell activation and a specific response^19^. It is so understood because the HLA molecules are directly implicated in the genetic control of the immune response.

We do not know of any Brazilian study that associates HLA type with RAS. Having said that, the goal of the present investigation was to typify classes I and II of HLA molecules from Brazilian patients with RAS and compare with the control group made up of 964 healthy Brazilians.

## MATERIALS AND METHODS

### Material

This is a prospective, cross-sectional, investigative study, carried out from February of 2004 through May of 2006. It was approved by the Ethics in Research Committee of the University of São Paulo Medical School, under protocol # 876/04.

### Study Group

The study group was initially made up of 58 patients with diagnostic suspicion of RAS, seen at the Stomatology Division of the ETN Clinic of the University of São Paulo Medical School Hospital.

### Control Group

The control group was made up by a selection of 4172 healthy Brazilian volunteers, members of the bone marrow donors data bank of the Transplant Center of the University of São Paulo Medical School Hospital.

From these, we selected only one member of each family, in order to avoid the presence of haplotypes, at the end making up a total of 962 Brazilian individuals.

### Method

The patients were submitted to an interview, general physical exam, complete otorhinolaryngological exam, and a previous workup protocol, besides biopsies of the lesion and adjacent mucosa.

The prior workup protocol involved fast glucose, protein electrophoresis, CBC, coagulogram (TP + TT + TTPA), anti-HIV 1 and 2, dosage of total complement and fractions, rheumatoid factor, antinucleus factors, serum dosage of IgA, IgG, IgM, VHS, C-Reactive protein and serology for syphilis.

In order to rule out the etiology of ulcers associated with other diseases, such as pemphigoid lesions, Behcet's disease, Crohn's disease or vasculitis of other nature, all the patients were submitted to lesion biopsy for later histopathology test and direct immunofluorescence test.

Biopsy was carried out under 2% xylocaine injection without vasoconstrictor, by means of a puncher the size of the lesion (4-5mm). Two samples were taken; one with the lesion and one with the healthy adjacent area, the former was placed in a flask with 10% formaldehyde and sent to histopathological exam. The second sample was placed on gauze soaked in saline solution and immediately referred to the Skin Immunology Division of the Department of Dermatology for direct immunofluorescence.

### Inclusion and Exclusion Criteria

In the study we included all those patients who reported at least on episode of aphtha (s) per month for a minimum period of one year, as well as those who signed the informed consent form agreeing with the procedures employed.

We excluded those patients who had alterations in the serum exam or those whom in the histopathology and direct immunofluorescence study had alterations that characterize other diseases. These patients were referred to specific treatment of the alterations found.

In order to obtain DNA, we collected 10ml of total peripheral blood in an EDTA 25mM flask. DNA extractions were carried out by the Hexadecyltrimethylammonium Bromide/dodecyltrimethylammonium Bromide (DTAB/CTAB) test and HLA class I and II types by chain polymerization - specific oligonucleotide sequence (PCR-SSO). The analysis was carried out in the Heart Institute of the University of São Paulo Medical School Hospital (Incor - FMUSP).

Blood collection, DNA extraction and HLA typing of both classes from the control group had already been carried out in this same lab.

### Statistical Analysis

In order to check whether or not there was any association between HLA antigens and their subgroups and RAS disease, we used the Chi-Squared test, and a given association was considered statistically significant when the test's description level (p) were lower than 0.05 (or 5%). In order to estimate the power of the associations found, we calculated the relative risks (RR) and their respective confidence intervals of 95% (CI 95%).

The data was analyzed using the Statistical Package for Social Sciences (SPSS® for Windows 10.0; SPSS Inc, Chicago, Illinois, USA) software.

## RESULTS

Of the 58 initial patients, 27 were taken off the study for numerous reasons. Of the remaining 31 patients, 17 (54.84%) were women, 14 (45.16%) were men. Age varied between 7 and 70 years, reaching an average of 36.14 years and standard deviation of 15.33 (Table 1).

**Table 1:** Demographic data from the 31 patients with RAS according to the type of RAS presented.

	Aphtha type				
	Minor	Major	Herpetiform	p	Total
	(n = 21)	(n = 10)	(n = 0)		(n = 31)
	(67,74 %)	(32,26 %)	(0,00 %)		(100,00 %)
Gender					
Male	10 (47,6 %)	4 (40,0 %)	0 (0,0 %)	1,0	14 (45,2 %)
Female	11 (52,4 %)	6 (60,0 %)	0 (0,0 %)		17 (54,8 %)
Age (years)	35,6 ± 14,5	37,2 ± 17,5	–	0,79	36,1 ± 15,2
< 20	2 (9,5 %)	2 (20,0 %)		0,35	4 (12,9 %)
20 a 39	12 (57,1 %)	3 (30,0 %)			15 (48,4 %)
≥ 40	7 (33,3 %)	5 (50,0 %)			12 (38,7 %)

As to the types of aphthas the study patients had, there was a predominance of the minor type (67.74%). Please notice the absence of patients with herpetiform aphthas (Table 1).

Regarding the frequency type of Class I an II HLA antigens in the study group, Table 2 shows the frequency of each patient.

**Table 2:** Distribution as to HLA class I and II frequency of 31 patients with RAS.

Patient	HLA-A	HLA-B	HLA-Dr
1	A1 A11	B35 B63	DR13 DR16
2	A23 A26	B50 B51	DR4 DR13
3	A24 A24	B35 B65	DR1 DR16
4	A25 A34	B7 B42	DR7 DR10
5	A33 A68	B65 B71	DR1 DR 17
6	A2 A2	B7 B61	DR10 DR11
7	A2 A32	B18 B35	DR4 DR16
8	A2 A66	B35 B58	DR13 DR17
9	A74 A74	B35 B81	DR9 DR14
10	A3 A29	B35 B44	DR8 DR7
11	A1 A24	B13 B56	DR1 DR15
12	A23 A80	B44 B72	DR4 DR7
13	A24 A33	B35 B49	DR7 DR15
14	A24 A29	B51 B64	DR7 DR11
15	A2 A23	B50 B51	DR7 DR11
16	A2 A30	B18 B39	DR14 DR17
17	A32 A66	B35 B58	DR4 DR11
18	A11 A11	B7 B35	DR15 DR103
19	A2 A24	B18 B35	DR8 DR17
20	A1 A2	B37 B50	DR7 DR103
21	A1 A3	B35 B57	DR11 DR17
22	A1 A23	B18 B81	DR7 DR11
23	A24 A31	B35 B51	DR11 DR13
24	A2 A29	B44 B64	DR7 DR17
25	A2 A3	B51 B62	DR13 DR16
26	A31 A33	B39 B65	DR1 DR8
27	A24 A30	B8 B35	DR11 DR17
28	A3 A33	B35 B49	DR7 DR7
29	A11 A33	B13 B35	DR15 DR15
30	A2 A33	B41 B65	DR1 DR17
31	A2 A24	B18 B62	DR8 DR15

Table 3 shows the prevalences of HLA Class I antigen in which there was a statistically significant difference between the study group (without considering the type of aphtha) and the control group. The relative risk (RR) of patients with RAS who had HLA frequencies of A33, B35 and B81 was of 3.48 (CI95% 1.38 – 8.8; p = 0.016); 3.48 (CI95% 1.69 – 7.16; p < 0.001) and 8.22 (CI95% 1.67 – 40.41; p = 0.036), respectively, times the control group.

**Table 3:** Class I HLA antigen frequency in patients with Recurrent Aphthoid Stomatitis (RAS) compared to the control group.

	Group			
	RAS (n = 31)	Control (n = 961)	p	RR (CI 95%)
A33	6 (19,4 %)	62 (6,5 %)	0,016	3,48 (1,38 – 8,80)
B35	15 (48,4 %)	204 (21,2 %)	< 0,001	3,48 (1,69 – 7,16)
B81	2 (6,5 %)	8 (0,8 %)	0,036	8,22 (1,67 – 40,41)

RR = Relative Risk; CI = Confidence Interval

Class II HLA frequencies of the study group which had greater index were Dr7, present in 10 patients (16.13%), Dr11 and Dr 17, the latter two were present in eight patients (12.90%). When compared to the control group, there was no statistically significant difference.

When RAS patients were broken down according to the type of aphtha, the prevalence of HLA A33 and B35 frequencies in the group with aphtha minor remained higher than those from the control group, in a statistically significant way, with RR of 3.27 (1.1 – 9.4; p = 0.047) and 4.73 (CI95% 2.0 – 11.1; p < 0.001), in relation to the control group, respectively, according to Table 4. Because of the reduction in the number of patients after the subdivision was made, the differences in HLA B81 frequency prevalences between both groups of aphtha and control patients, and HLA A33 and B35 between the aphtha major and control groups have a greater likelihood (5%) of being by chance and, therefore, not statistically significant (p > 0.05).

**Table 4:** Class I HLA antigen frequency in patients with major and minor Recurrent Aphthoid Stomatitis compared to the Control Group.

	Group			
	Aphtha Minor (n = 21)	Controls (n = 961)	p	RR (CI 95%)
A33	4 (19,0 %)	62 (6,5 %)	0,047	3,27 (1,1 – 9,4)
B35	12 (57,1 %)	204 (21,2 %)	<0,001	4,73 (2,0 – 11,1)
B81	1 (4,8 %)	8 (0,8 %)	0,177	5,41 (0,8 – 36,1)
	Group			
	Aphtha Major (n = 10)	Controls (n = 961)	p	RR (CI 95%
A33	2 (20,0 %)	62 (6,5 %)	0,137	3,54 (0,8 – 16,3)
B35	3 (30,0 %)	204 (21,2 %)	0,452	1,58 (0,4 – 6,1)
B81	1 (10,0 %)	8 (0,8 %)	0,089	11,88 (1,7 – 84,2)

RR = Relative Risk; CI = Confidence Interval

## DISCUSSION

Recurrent aphthoid stomatitis is a common disease in the oral cavity and its etiology is still undefined^20^,^3^,^7^. It may be associated with local and systemic factors, its origin is immunological, microbiological and genetic^21^,^22^.

According to Jurge et al.^4^, the incidence of RAS is more common in the second decade of life, between 20 and 30 years of age. In our study, the incidence was also higher in this same age range, with a total of eight patients (25.80%). Values very near this one, seven (22.5%), were obtained in the two following age ranges (30 to 40 years and 40 to 50 years).

Females had the higher RAS prevalence, 54.84%, when compared to males, 45.16%. These data are in agreement with the findings by Axéll and Henricsson23, but are also in conflict with those found by Miller et al.^9^, in which males had a higher prevalence.

In our study, the HLA frequencies that had the highest rates of patients with RAS were: HLA-A33, HLA-B35 and HLA-B81. When seen under the light of disease subtype, we found significant values for aphtha minor, in the frequencies of HLA-A33 and HLA-B35. For RAS Major, no frequency presented statistically significant value.

In an English population, Challacombe et al.^18^ found the HLA-B12 frequence associated with patients with RAS. When analyzed in relation to the subtypes of aphthoid Stomatitis the same B12 frequency was statistically significant only for herpetiform aphthas. For types Major and Minor, no prevalent frequency was found by the authors. In our study, because of its low incidence, it was not possible to find patients with the herpetiform type.

The same HLA-B12 frequency was found by Malmström et al.^11^ when they studied 14 Finish patients with RAS. Differently from our studies, these authors did not use a control group, and their RAS patient sample was small.

Gallina et al.^12^, with a sample of 26 Sicilian patients with RAS, found a statistically increased value for HLA-DR7 frequency. As we make a critical analysis of the experimental group selected, we notice that patients with only two episodes per year were considered RAS patients, and such characteristic did not match the RAS clinical picture established by Stanley^5^ and Scully^2^, who characterized as RAS those monthly episodes of aphtha for at least one year, the same we did in our study.

Albanidou-Farmaki et al.^14^ did a study with 106 patients with RAS. The HLA-DR5 frequency, differently from our findings, was predominant in these patients. On the other hand, the HLA-DR4 frequency was reduced.

Sun et al.^24^ stated that the HLA-DRw9 frequency can be considered a genetic marker for RAS in the Chinese population. Their study was made up of 80 Chinese patients with RAS. In this study, they typed the HLA-DR, differently from our study, in which we also typed the HLA-A and B.

In an Arab population, Jaber et al.^25^ studied 22 patients with RAS, and observed a statistically significant increase in HLA-B52 and B44 molecules in patients with this disease when compared to the control group. The HLA-B51 frequency has been found in some papers associated with RAS in countries like Israel and Korea^26^,^27^.

It is important to stress that with the progress in HLA typing methodology, the serum method is no longer used, in which it was necessary to have specific ligants (antibodies) in order to show the presence of the corresponding HLA frequence, as used by some authors^11^,^12^,^14^,^18^,^28^,^29^. In our study, we used the cellular method, in which one uses the mixed lymphocitic reactions, making the HLA typing more reliable, allowing the discovery of new frequencies^30^.

On the other hand, according to Braun Prado et al.^31^ and Monte et al.^32^, the HLA-B35 frequency is extremely common in the general Brazilian population, and such fact was also seen in our control group. Nonetheless, it is important to stress that although the HLA-B35 frequency was also found high in our experimental group; such fact did not happen by chance, there was a statistically significant difference in the incidence of this gene between the two groups.

We must also remember that, as mentioned by Louzada-Junior et al.^33^ the reduced frequency of HLA typing could be a protection factor for RAS. In our study we did not notice any of such events.

Finally, the fact that many papers found HLA frequencies for RAS different from the ones found in our study, probably happened because the populations studied were from different nationalities (Danish, Scottish, Israelis, Chinese, Koreans) and ethnic origins different from ours, a highly mixed races country.

Our findings show that the HLA frequencies must be done specifically for each population, so that we can map the possible genetic factors involved in the genesis of this disorder.

We have to stress in our study that the lack of patients with herpetiform recurrent aphthoid stomatitis and the low number of patients with stomatitis type major, prevented a more accurate assessment of the HLA frequencies associated with these disease forms.

At any rate, the relatively reduced series of patients with RAS in our study does not invalidate our results, having seen that the HLA frequencies found in our patients were very different from those obtained from the control group of patients without RAS.

## CONCLUSION

The results from our study suggest that minor RAS is associated with the HLA-A33 and HLA-B35 frequencies in the Brazilian population.

## ACKNOWLEDGEMENT

This paper counted on the financial support from the FAPESP - Fundação de Amparo a Pesquisa do Estado de São Paulo - FAPESP (Process # 05/51085-0).
